# Health impacts caused by excessive sodium consumption in Brazil: results of the GBD 2019 study

**DOI:** 10.1590/0037-8682-0266-2021

**Published:** 2022-01-28

**Authors:** Larissa Fernanda Fonseca Guedes, Mariana Santos Felisbino-Mendes, Aline Siqueira Fogal Vegi, Adriana Lúcia Meireles, Mariana Carvalho de Menezes, Deborah Carvalho Malta, Ísis Eloah Machado

**Affiliations:** 1 Universidade Federal de Ouro Preto, Programa de Pós-Graduação em Saúde e Nutrição, Ouro Preto, MG, Brasil.; 2 Universidade Federal de Minas Gerais, Programa de Pós-Graduação em Enfermagem, Belo Horizonte, MG, Brasil.; 3 Universidade Federal de Ouro Preto, Departamento de Nutrição Clínica e Social, Ouro Preto, MG, Brasil.; 4 Universidade Federal de Ouro Preto, Departamento de Medicina de Família, Saúde Mental e Coletiva, Ouro Preto, MG, Brasil.

**Keywords:** Risk factors, Sodium, Dietary, Noncommunicable diseases, Global Burden of Disease, Nutritional Epidemiology

## Abstract

**INTRODUCTION:**

Excessive sodium consumption is associated with increased blood pressure, which is an important risk factor for non-communicable diseases (NCDs). This study therefore aimed to describe the burden of NCDs attributable to excessive sodium consumption among Brazilians.

**METHODS:**

This observational study used mortality and Disability Adjusted Life Years (DALY) rates*,* and their respective uncertainty intervals (UI), from the Global Burden of Disease Study 2019 (GBD 2019)*.* The burden was obtained by the population attributable fraction of each NCD, considering the minimum theoretical value of risk (intake of 0-3g of sodium/day); the excessive consumption proportion in the population, obtained through population inquiries; and the relative risks obtained through meta-analyses.

**RESULTS:**

Excessive sodium consumption was the third highest dietary risk contributing to deaths (30,814; 95% UI = 2,034 - 84,130) and DALYs (699,119; 95% UI= 43,130 - 1,914,066) in 2019. States from the Northeast region had the highest age-standardized rates of deaths and DALYs, and the male population was more affected by NCDs caused by excessive sodium consumption. Cardiovascular diseases were the main contributing factors in the burden attributable to excessive sodium consumption.

**CONCLUSIONS:**

Regardless of the progress in addressing NCDs related to this risk factor, the impact remains high, especially among men and in the Northeast region. More effective measures are needed to reduce sodium in industrialized products, such as health promotion actions to combat sodium consumption, in order to prevent and control NCDs in Brazil.

## INTRODUCTION

Non-communicable diseases (NCDs) include a wide range of illnesses responsible for 76% of all deaths in Brazil, with cardiovascular diseases (CVD) being the most prevalent cause of morbimortality in the country^1^ ^,^ ^2^. More than causing a decline in life quality and financial losses to families, such diseases also contribute to increased spending with medical assistance and welfare^3^.

Among a variety of unhealth dietary habits, evidence shows that the excessive sodium consumption is connected to stomach cancer and chronic kidney disease^4^ ^,^ ^5^, as well as to a progressive increase in blood pressure, which also contributes to a higher risk of CVD^6^ ^,^ ^7^. By contrast, a reduction in sodium consumption contributes to blood pressure control, consequently helping to control CVD, which in turn proves the importance of this risk factor in the context of these diseases^8^.

The World Health Organization establishes that the daily sodium consumption should not be higher than 3g, the equivalent of 5g of common salt^7^. However, according to the National Health Survey (NHS) in 2013, Brazilians consumed almost twice (9.34g of salt/day). Moreover, 97.6% of adults presented consumption above the recommended amount^9^ ^,^ ^10^, which constitutes an impact on health. The prevalence of hypertension in Brazilian adults was 32.3%, according to the NHS, which used the diagnostic criteria of measuring blood pressure and medication use^11^. 

In this regard, we aimed to describe the burden of NCDs on Brazilians’ health, attributed to the excessive sodium consumption. This study will contribute to understanding the real impact of this risk factor, since this process involves a robust methodological approach that is rarely seen in Brazil. The estimates of the burden of NCDs for the country and its 26 states and one federal district will enable the identification of regional inequalities and may support the proposal of policies and actions to promote healthy eating based on evidence, which are more appropriate for regional realities. This will also favor investments in the optimization of the Brazilian Unified Health System (SUS, in Portuguese). 

## METHODS

This is an observational and ecological study, using estimates taken from the GHDx portal of the Institute for Health Metrics and Evaluation (IHME) from the University of Washington, which conducted the Global Burden of Disease Study 2019 (GBD 2019) (http://ghdx.healthdata.org/). The database is a public domain and does not contain individual information. Therefore, there was no need for approval by a Research Ethics Committee. 

Mortality rates were used to analyze the burden of the NCDs attributable to the excessive sodium consumption, together with the main measurement of the GBD 2019, the Disability Adjusted Life Years (DALYs), allowing for the simultaneous measurement of the impact of premature mortality and disabilities that affect the quality of life of a given population.

Considering GBD 2019 methodology, four key components are needed to produce the estimates: 1) the levels of exposure to the risk factor - excessive sodium consumption, more than 3g/day; 2) the relative risk (RR) for an undesirable outcome due to exposure; 3) the theoretical minimum risk exposure level (TMREL) - consumption of sodium equal to or less than 3g/day; and 4) the measurement of the burden of disease (number of deaths or DALYs)^5^.

The level of consumption considered adequate is that in which the risk for any disease is minimum, according to scientific literature review^12^. Therefore, the TMREL is used to calculate the burden of disease that could be avoided if, in the past, the population exposure had been sustained at the theoretical minimum risk of exposure. In this case, the minimum risk associated with sodium consumption corresponded to the ingestion of up to 3g per day, estimated by the 24-hour urinary sodium excretion^5^.

To estimate the ingestion of this nutrient by the Brazilian population, the GBD 2019 uses literature reviews, data from national surveys conducted by the Secretariat of Health Surveillance (SVS, in Portuguese) from the Brazilian Ministry of Health, and Brazilian Statistics Institute (IBGE, in Portuguese), such as Household Budget Surveys (POF 2008-2009, in Portuguese) and the Brazilian Surveillance System of Risk Factors for Chronic Disease through Telephone Interviews^13^. In the absence of local data to calculate the estimates, international sources can be used by the GBD^5^.

Each source used can be affected by bias; therefore, adjustments were used, considering for sodium, the 24-hour urinary excretion as the “gold standard”. Hence, to make sources that used different gold standard methods comparable, this study applied a network meta-regression (MR-BRT). Afterwards, the estimate for the daily average ingestion of sodium according to age, sex, country, and year, equivalent to the proportion of population risk, was modeled by spatial-temporal Gaussian process regression (ST-GPR)^5^.

To estimate the attributable fractions, the GBD group selected the risk-outcome pairs by research in the available literature, aimed at identifying evidence that supports the causality relation between the excessive sodium consumption and the health outcomes, and to obtain the relative association and uncertainty risks^5^. Besides the direct effect of excessive sodium consumption on health outcomes, there is evidence that excessive sodium consumption is associated with an increase in blood pressure^6^ ^,^ ^14^, which is considered an intermediate risk factor by the GBD 2019 study. Hence, the effects of sodium on cardiovascular outcomes and chronic kidney disease are considered to be mediated by hypertension^12^.

The evidence used by the GBD 2019 was the basis to establish that the increase of 1g in sodium consumption, of more than 3g/day, is enough to increase systolic blood pressure in 10 mmHg. The TMREL for hypertension adopted by the GBD 2019 is based on cohort study prospects, which have shown an increase in the risks of mortality and loss of health in cases of systolic blood pressure of higher than 110-115 mmHg. It has also been observed that, for each 10 mmHg above the TMREL, there is an increase in RR^5^.

Table 1 lists the diseases, which are attributed directly to the excessive sodium consumption and mediated by hypertension, according to codes used by the International Statistical Classification of Diseases and Related Health Problems (ICD-10). After these definitions, it is possible to obtain the Population Attributable Fraction (PAF), which corresponds to the proportion of the outcomes attributed to excessive sodium consumption. In other words, the PAF in this case represents the reduction in a given measurement of health (death or DALY, for instance) within a given period, if the consumption of sodium in the past had been reduced to 0-3g per day, per inhabitant^15^. Finally, the estimate of the burden attributable to excessive sodium consumption was obtained by multiplying the PAF by the total of DALYs or deaths for each cause associated with this risk factor. 

**TABLE 1:** ICD-10 codes for diseases attributed to the excessive consumption of sodium, GBD 2019.

DISEASE	ICD-10
**Direct effect**	
Stomach cancer	C16-C16.9, Z12.0, Z85.02-Z85.028
**Effect mediated by hypertension**	
Rheumatic heart disease	I01-I01.9, I02.0,I05-I09.9
Ischemic heart disease	I20-I21.6, I21.9-I25.9, Z82.4-Z82.49
Stroke	G45-G46.8, I60-I62, I62.9-I64, I64.1, I65-I69.998, Z82.3
Hypertensive heart disease	I11-I11.2, I11.9
Non-rheumatic valvular	I35-I35.9
Cardiomyopathy and myocarditis	I42.0-I42.5, I42.7
Atrial fibrillation and flutter	I48-I48.92
Aortic Aneurysm	I71
Peripheral artery disease	I70.2-I70.92, I73-I73.9
Endocarditis	B33.21, I33-I33.9, I38-I38.0, I39-I39.9
Other cardiovascular and circulatory disease*	
Chronic kidney disease	I12-I13.9

*This aggregate cause incorporates less common cardiovascular diseases that are not modelled independently.

In the GBD 2019 study, 95% uncertainty intervals (95% UI)^16^ are calculated for all the results that provide information about the variability of estimates resulting from errors due to the sampling process and non-sampling errors caused by adjustments in data sources and modelling. The intervals correspond to 2.5 and 97.5 percent of the values obtained after the repetition of 100 draws for each estimate, after each step of the estimation process, using the Monte Carlo simulation method^17^_._

In the present study, the DALY and death measurements are presented according to sex and state, in its crude format, and age-standardized, using GBD 2019 global population as the main reference. Comparisons were made by age groups, sex, and Brazilian states. 

## RESULTS

**FIGURE 1:**
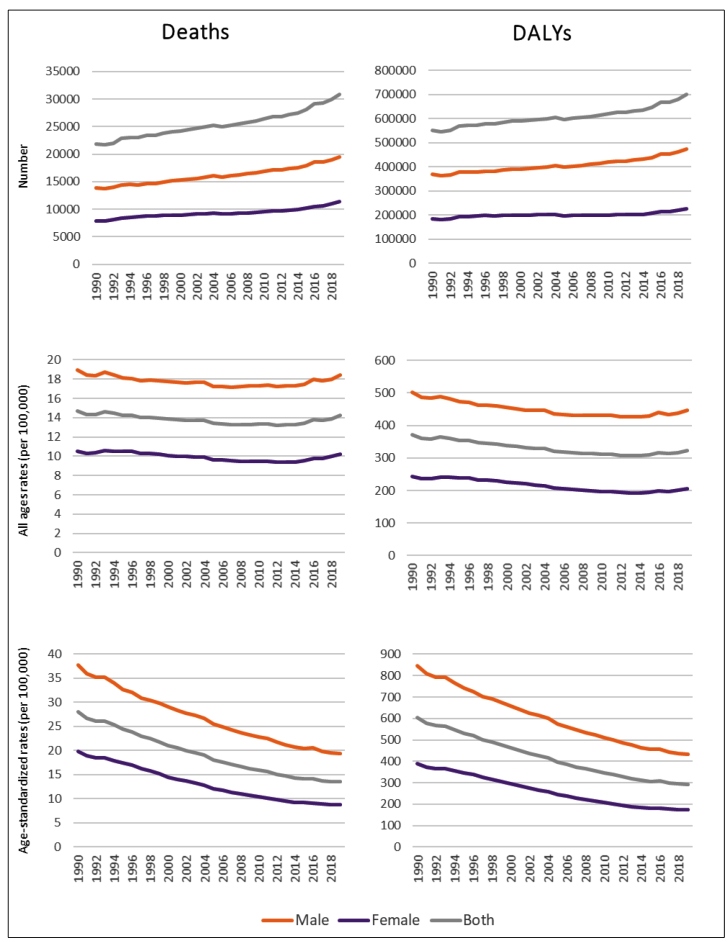
Total number and rates of deaths and DALYs attributable to the excessive consumption of sodium, according to sex, Brazil, 1990 and 2019.

In 2019, the excessive sodium consumption was responsible for 30,814 deaths (95% UI: 2,034 - 84,130), which was considerably higher among men (19,480 deaths; 95% UI: 1,413 - 49,738) than among women (11,334 deaths; 95% UI: 506 - 34,785). This risk factor also caused 699,119 DALYs (95% UI: 43,130 - 1,914,066); the number of DALYs for males was more than double (472,367 DALY; 95% UI: 33,074 - 1,187,175) the number for females (226,752 DALY; 95% UI: 10,355 - 698,892). The numbers observed in 2019 were higher than those from 1990, when the excessive sodium consumption was responsible for 21,830 (95% UI: 1,240 - 58,830) deaths and 551,373 (95% UI: 30,752 - 1,485,468) DALYs in Brazil. In terms of the changes that occurred between 1990 and 2019, there was a small variation in the gross rates and a decrease in the age-standardized rate (Figure 1).

**FIGURE 2:**
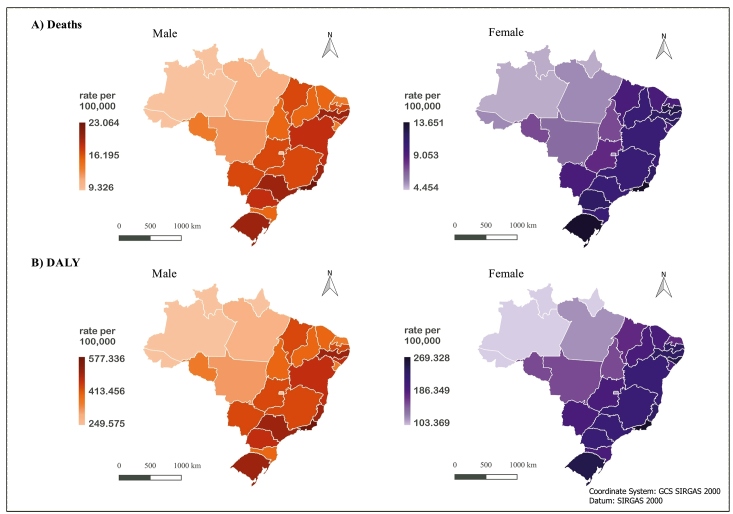
Mortality rates and DALYs attributable to the excessive consumption of sodium per 100,000 inhabitants, for all age groups, according to sex and Brazilian states, Brazil, 2019.

For Brazilian states, the 2019 mortality rates for all ages attributable to excessive sodium consumption for the males varied from 9.32 per 100,000 inhabitants (95% UI: 0.47 - 24.87) in Amapá to 23.06 per 100,000 inhabitants (95% UI: 1.17 - 59.27) in Rio de Janeiro, where the rate as 2.47-fold higher. By contrast, the variation of mortality for females was from 4.45 per 100,000 inhabitants (95% UI: 0.18 - 13.94) in Roraima to 13.65 per 100,000 inhabitants (95% UI: 0.57 - 41.40) in Rio Grande do Sul, where the rate was 3.7-fold higher. Concerning the DALY rate for NCDs attributable to the excessive sodium consumption for men, this value varied from 249.57 per 100,000 inhabitants (95% UI: 12.83 - 666.13) in Amapá to 577.33 per 100,000 inhabitants (95% UI: 28.40 - 1,442.53) in Rio de Janeiro. Meanwhile, the DALY rates for women ranged from 103.36 per 100,000 inhabitants (95% UI: 4.59 - 329.26) in Amazonas to 269.32 per 100,000 inhabitants (95% UI: 11.64 - 845.91) in Rio de Janeiro. The rates of mortality and DALYs were considered higher for the males than for the females (Figure 2). 

**FIGURE 3:**
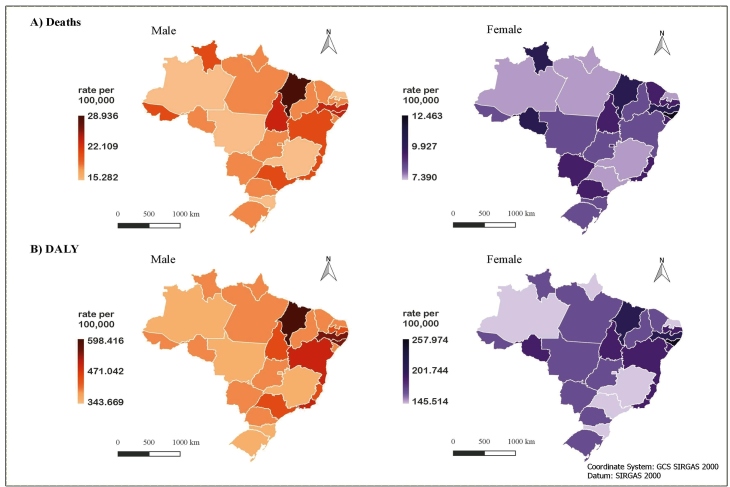
Age-standardized mortality rates and DALYs attributable to the excessive consumption of sodium, according to sex and Brazilian states, Brazil, 2019.

For the standardized rates attributable to the excessive sodium consumption, a variation was noted in mortality rates, going from 15.28 per 100,000 inhabitants (95% UI: 0.83 - 39.98) in Minas Gerais to 28.93 per 100,000 inhabitants (95% UI: 1.61 - 76.82) in Maranhão among males in 2019. Among the females, the variation was from 7.39 per 100,000 inhabitants (95% UI: 0.31 - 23.37) in Amazonas to 12.46 per 100,000 inhabitants (95% UI: 0.49 - 37.03) in Alagoas. For DALYs, the standardized rates of NCDs attributable to the excessive sodium consumption varied for males, from 343.66 per 100,000 inhabitants (95% UI: 19.90 - 884.00) in the Federal District to 598.41 per 100,000 inhabitants (95% UI: 32.46 - 1,589.67) in Maranhão in 2019. For females, the DALY rate ranged from 145.51 per 100,000 inhabitants in Amazonas (95% UI: 6.34 - 464.25) to 257.97 per 100,000 inhabitants (95% UI: 10.63 - 790.59) in Alagoas. The standardized mortality rates and the DALY rates were also substantially higher for men than for women in all Brazilian states (Figure 3). For detailed information about Figures 2 and 3, see the Supplementary Data.

In conclusion, in terms of the non-standardized rates, the states of the South, Southeast, and Northeast had the highest mortality and DALY rates associated with the excessive sodium consumption, while those in the North had the lowest, for both sexes, in 2019. Once the rates were age-standardized, higher death rates were observed in the Northeast, especially in Maranhão and Alagoas. Minas Gerais and Mato Grosso had the lowest death rates, while the Federal District and Mato Grosso had the lowest DALY rates for males; however, Amazonas still presented the lowest death rates and DALY rates for females.

**FIGURE 4:**
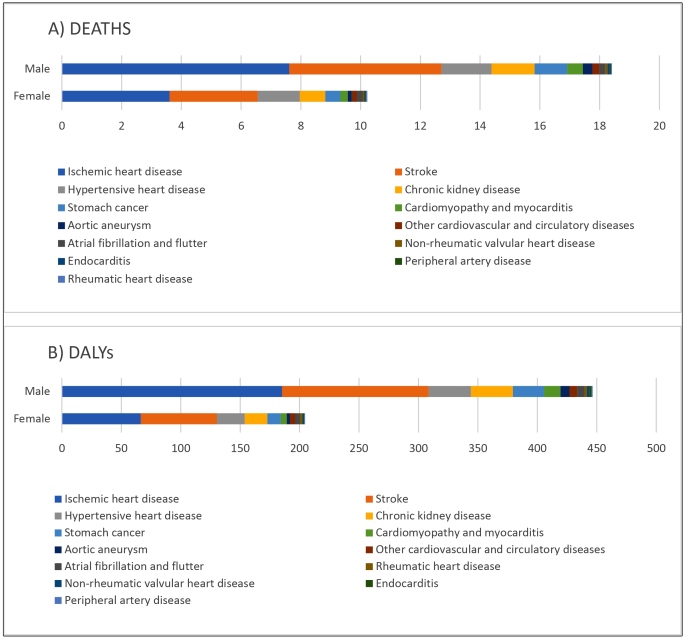
Mortality rates and DALYs attributable to the excessive consumption of sodium per 100,000 inhabitants, according to cause and sex, Brazil, 2019.

In 2019, the main NCDs related to excessive sodium consumption were CVDs, especially ischemic heart disease, stroke, and hypertensive heart disease. These were followed by chronic kidney disease and stomach cancer. In both sexes, ischemic heart disease and stroke had the highest death rates and DALYs compared to other diseases (Figure 4). 

## DISCUSSION

Between 1990 and 2019, the number of deaths and DALYs caused by excessive sodium consumption increased, which now stands out as an important dietary risk factor in Brazil. States from the South, Southeast, and Northeast have a higher burden of NCDs related to an excessive sodium consumption, when compared to the rest of the country. However, when the rates were age-standardized, the states from the Northeast, such as Maranhão, Alagoas, and Pernambuco, stood out in terms of deaths and DALYs attributable to this risk. Among the main NCDs that affect the Brazilian population due to the excessive sodium consumption, one can highlight CVDs, such as cardiac ischemia, stroke, and hypertensive heart disease. 

Moreover, men have a higher burden of NCDs caused by that factor than do women. From 1990 to 2019, although there was an increase in the absolute numbers of deaths and DALYs attributed to the excessive sodium consumption, the gross rates show a tendency of stability, with a decrease in standardized rates starting in 1990. This result indicates that the increase in the number of deaths and DALYs is most likely related to population growth and aging, and not to an increase in exposure to excessive sodium consumption. 

Household Budget Surveys (HBS) data shows that in 2008-2009, 89% of men and 70% of women between 19 and 59 years of age consumed more than the 2,300mg sodium limit, while 80% of men and 62% of women over 60 consumed more than the limit^18^. However, in 2017-2018, the proportional population that consumed more than recommended amount was smaller (53.5%), which was higher in adult males (74.2%) and lower in older female adults (25.8%)^19^.

The reduction in excessive sodium consumption in the country was the effect of several actions implemented during the period. In 2001, the HiperDia was created, a program which promotes strategic action in Primary Health Care for the control of diabetes mellitus and hypertension, and to promote improvements in patients’ life quality^20^. With closer monitoring, the study showed that 70% of the HiperDia patients had received information about controlling sodium levels in their diet^21^. However, that program is limited to the assistance area and serves people who already have hypertension.

In 2011, the Ministry of Health launched the 2011-2022 Strategic Action Plan for Tackling NCDs^22^. One of the targets was to reduce the average salt consumption to 5g per day, promoting intersectoral actions, such as voluntary agreements with the food industry, aimed at reducing the sodium level in 35 food categories^23^. However, while agreements with the industry remain lenient, data from the HBS show that the calories from processed foods have increased in recent decades, from 12.6%^24^ to 18.4%^25^, consequently increasing sodium consumption. By contrast, in the United Kingdom, similar agreements covered approximately 80 foods categories and included stricter targets^26^. Deficient monitoring is another limitation that possibly contributes to the fail to achieve the targets^27^ ^-^ ^29^. Therefore, voluntary agreements may not be enough to reach the targets for 2022^30^. However, to reduce the alarming number of deaths and DALYs caused by the excessive sodium consumption, public policies must become more intensified and bolder^29^.

Besides maintaining the continuous monitoring of industrialized products available in the market, measures that encourage and facilitate access to foods *in natura*, like the incentive to family agriculture, are necessary^21^. Other strategies that must be reinforced are education campaigns, which seek to encourage a more careful reading of labels and make the population more aware of the dangers of excessive sodium consumption^31^, especially about the addition of table salt and condiments, the main sources of sodium for Brazilians^32^.

Regarding sex, as men consume more calories, it is expected that they also consume more micronutrients, including sodium. Otherwise, several studies demonstrate that healthy eating markers are more prevalent among women^33^ ^,^ ^34^. Women also seek out medical assistance more often and follow prescribed treatment more accurately when compared to men^35^. Moreover, factors determined by sex are possibly related to hormonal differences, which make women more sensitive to blood pressure reduction with less reduction in the sodium consumption than men^36^. Furthermore, historically, caring for eating is a domestic and feminine issue. Meanwhile, men, due to the pressures of work and the need to eat out of the house, end up seeking less healthy foods, which are typically found in snack bars and fast food restaurants^37^. Such foods have a higher number of calories as well as a higher sodium content^38^.

 The findings of this study using the GBD estimates are coherent with data from national studies which verified not only the more frequent, excessive sodium consumption among males^39^ ^-^ ^41^, but also a lower level of awareness and control of blood pressure. This represents an important challenge for improving the quality and life expectancy of men^42^ ^,^ ^43^.

Health promotion policies compatible with regional realities would also be an efficient way of minimizing the impact of NCDs attributed to an excessive sodium consumption. Brazil is the country with the 6th biggest population in the world, its dimensions are continental, and the rates of morbimortality are intricately linked to geographic, economic, and social inequalities^44^. In this context, considering the gross rates, we can see that Rio de Janeiro is the state with the highest burden of NCDs attributed to the excessive sodium consumption, followed by Rio Grande do Sul. Meanwhile, states from the North, like Amapá, Roraima, and Amazonas had the lowest burdens. 

These results may be related to the large proportion of older adults in those states, an age group most likely to have hypertension, and more susceptible to its aggravating effects, such as CVDs^45^. Meanwhile, the North region, in 2019, had a higher proportion of younger age groups, nearly 43% of the region’s population is under 25 years^46^. Furthermore, the 2013 NHS verified that sodium consumption was lower in the North (8.78 g/day 95% CI 8.68 - 8.8) and higher in the Southeast (9.50 g/day; 95% CI 9.36 - 9.64) and in the South (9.40 g/day; 95% CI 9.25 - 9.55). The prevalence of hypertension was higher, specifically in Rio de Janeiro, in the Southeast, and in Rio Grande do Sul, in the South^10^.

However, when age-standardized, the highest death and DALY rates attributed to the excessive sodium consumption in 2019 were identified in the Northeast’s states. Even though half of all Brazilian homes are registered in the family health units, and considering that the Northeast shows the highest percentage of registry^46^, only 65% of the hypertensive patients who had some kind of contact with health services were aware of their condition and only 33% had their blood pressure controlled^47^.

Hypertension is often a silent condition, and a lack of knowledge about its risk factor contributes to its under-diagnosis^48^. Therefore, deficiencies in the health system, combined with the low education level of a major part of the Northeast’s population^49^, contributes to a greater prevalence, since the lack of education is associated with difficulties in controlling hypertension successfully^47^.

Therefore, health education actions, and that which can bring the patient closer to the family health units, may reduce the geographic and economic barriers that compromise access to primary care and improve its quality, also improving the follow-up of the patients’ conditions^50^. Such actions can reduce the need for hospitalization and reduce CVD mortality, which can be avoided and are treatable at the primary care level^50^. However, individual dietary control alone is not enough to minimize the high blood pressure effects, as there is also mandatory intersectoral strategies that facilitate access to *in natura* foods and that reduce the consumption of industrialized foods and their sodium content. 

One important limitation of this study is related to the multiple data sources used to estimate sodium intake in Brazil, which can contain specific biases. In addition, data scarcity on estimated sodium intake for the Brazilian population contributed to the amplitude of the uncertainty intervals presented. Thus, it is noted that although there are differences in mortality rates and DALYs by sex and state, caution is needed in interpreting the results, since the uncertainty intervals are quite wide and often overlap. Moreover, the sources used by the GBD 2019 do not include data gathered recently, such as the laboratory results from the NHS 2013 and the intake data from HBS, 2018-2019, which reflect the Brazilian context more accurately. We hope that the next study versions will include these two important sources, thereby enabling a reduction in the statistical uncertainty in the estimates of exposure to excessive sodium consumption and of the burden of diseases attributed to it in Brazil^12^. 

On the other hand, the GBD study complements other studies in the field of epidemiology that investigate the determining factors of health outcomes, generating methodological solutions to produce estimates of the burden of disease and injury attributable to risk factor comparable for the different countries with the application of the PAF^51^. Thus, the findings presented may lead to a better understanding of the impact of excessive sodium consumption as a risk factor for the population's health in Brazil, related to a substantial number of deaths and DALYs by NCDs, highlighting the need for investment in measures to prevent its consequences. 

Burden of disease estimates contribute to the evaluation of the magnitude of the impact of excessive sodium consumption on population health by applying the FAP measure. This knowledge can be used in the implementation of measures to prevent and control this risk factor, since it is related to the important share of deaths and disabilities from NCDs in Brazilian states. Therefore, it becomes necessary to propose policies that are more appropriate to regional realities, which favor the optimization of investments in SUS and decrease inequalities among states. Finally, it is necessary to promote massive campaigns that encourage the reduction of sodium consumption, especially among the male population, as well as campaigns that strengthen this recommendation in primary care.
