# Supplementary material for: Health impacts caused by excessive sodium consumption in Brazil: results of the GBD 2019 study
Source: Rev Soc Bras Med Trop. 2022 Jan 28;55(Suppl 1):e0266-2021. doi: 10.1590/0037-8682-0266-2021 (PMC9022945; doi:10.1590/0037-8682-0266-2021)
Supplement: Supplementary file 1 [file 1678-9849-rsbmt-55-e0266-2021-supp1.pdf]

1 - Death rates for all ages per 100,000 inhabitants attributable to excessive sodium consumption, male. Brazil, 2019 (95% UI).

				95% UI	
**Measure**	**Sex**	**Location**	**Value**	**Upper**	**Lower**
Deaths	Male	Brazil	18.412	47.012	1.336
Deaths	Male	Pará	12.461	32.632	0.680
Deaths	Male	Mato Grosso do Sul	17.332	45.103	0.967
Deaths	Male	Espírito Santo	20.157	51.720	1.106
Deaths	Male	Maranhão	18.924	50.136	1.089
Deaths	Male	Minas Gerais	17.053	44.092	0.887
Deaths	Male	Rio de Janeiro	23.064	59.270	1.175
Deaths	Male	Amapá	9.326	24.879	0.475
Deaths	Male	Piauí	17.373	44.764	0.966
Deaths	Male	Mato Grosso	13.372	35.053	0.786
Deaths	Male	Ceará	16.642	45.068	0.874
Deaths	Male	Paraíba	18.782	48.498	1.092
Deaths	Male	Pernambuco	20.376	52.594	1.270
Deaths	Male	Tocantins	17.351	46.672	0.958
Deaths	Male	Rondônia	14.650	38.181	0.787
Deaths	Male	Alagoas	18.343	47.892	0.995
Deaths	Male	Roraima	10.317	26.404	0.576
Deaths	Male	Sergipe	15.054	40.685	0.800
Deaths	Male	Acre	10.817	28.645	0.584
Deaths	Male	Goiás	16.626	43.340	0.931
Deaths	Male	Rio Grande do Sul	21.085	53.706	1.122
Deaths	Male	Rio Grande do Norte	16.223	44.058	0.928
Deaths	Male	Bahia	19.168	50.248	1.066
Deaths	Male	Paraná	19.835	52.292	0.972
Deaths	Male	Amazonas	10.216	27.999	0.593
Deaths	Male	São Paulo	20.370	49.677	1.235
Deaths	Male	Santa Catarina	16.582	43.642	0.894
Deaths	Male	Distrito Federal	10.453	27.125	0.582

2 - Death rates for all ages per 100.000 inhabitants attributable to excessive sodium consumption, female. Brazil, 2019 (95% UI).

				95% UI	
Measure	Sex	Location	Value	Upper	Lower
Deaths	Female	Brazil	10.222	31.376	0.457
Deaths	Female	Pará	6.069	19.059	0.254
Deaths	Female	Mato Grosso do Sul	9.293	28.689	0.389
Deaths	Female	Espírito Santo	11.052	34.475	0.461
Deaths	Female	Maranhão	9.046	27.925	0.389
Deaths	Female	Minas Gerais	10.176	31.108	0.429
Deaths	Female	Rio de Janeiro	13.400	41.646	0.570
Deaths	Female	Amapá	4.496	14.201	0.196
Deaths	Female	Piauí	10.398	33.054	0.433
Deaths	Female	Mato Grosso	6.941	21.032	0.289
Deaths	Female	Ceará	9.872	30.257	0.406
Deaths	Female	Paraíba	11.904	36.572	0.500
Deaths	Female	Pernambuco	11.223	35.209	0.475
Deaths	Female	Tocantins	7.958	24.651	0.334
Deaths	Female	Rondônia	7.579	23.477	0.310
Deaths	Female	Alagoas	11.343	33.640	0.455
Deaths	Female	Roraima	4.454	13.943	0.188
Deaths	Female	Sergipe	9.282	28.158	0.377
Deaths	Female	Acre	5.394	16.461	0.228
Deaths	Female	Goiás	8.735	26.754	0.377
Deaths	Female	Rio Grande do Sul	13.651	41.405	0.572
Deaths	Female	Rio Grande do Norte	9.479	28.789	0.373
Deaths	Female	Bahia	10.182	32.277	0.404
Deaths	Female	Paraná	11.133	34.058	0.459
Deaths	Female	Amazonas	4.792	15.341	0.206
Deaths	Female	São Paulo	10.351	32.153	0.448
Deaths	Female	Santa Catarina	10.030	29.993	0.416
Deaths	Female	Distrito Federal	6.116	18.947	0.255

3 - DALYs rates for all ages per 100.000 inhabitants attributable to excessive sodium consumption, male. Brazil, 2019 (95% UI).

				95% UI	
Measure	Sex	Location	Value	Upper	Lower
DALYs	Male	Brazil	446.476	1122.105	31.262
DALYs	Male	Minas Gerais	415.544	1083.469	21.177
DALYs	Male	Pernambuco	507.395	1312.495	29.118
DALYs	Male	Acre	270.323	710.351	14.277
DALYs	Male	Santa Catarina	402.750	1026.667	21.158
DALYs	Male	Alagoas	457.785	1193.823	24.079
DALYs	Male	Amazonas	253.764	694.369	14.390
DALYs	Male	Mato Grosso	342.110	875.159	19.140
DALYs	Male	Paraíba	434.739	1091.587	25.901
DALYs	Male	Maranhão	438.302	1150.704	23.861
DALYs	Male	Rio Grande do Norte	380.350	1019.270	21.508
DALYs	Male	São Paulo	498.316	1210.189	29.200
DALYs	Male	Amapá	249.575	666.139	12.835
DALYs	Male	Roraima	270.861	675.733	14.115
DALYs	Male	Mato Grosso do Sul	426.715	1086.883	24.361
DALYs	Male	Pará	308.372	807.241	15.529
DALYs	Male	Sergipe	365.065	969.437	20.074
DALYs	Male	Rio Grande do Sul	489.216	1227.609	25.034
DALYs	Male	Tocantins	400.803	1048.856	21.042
DALYs	Male	Rio de Janeiro	577.336	1442.539	28.402
DALYs	Male	Espírito Santo	488.938	1245.803	25.561
DALYs	Male	Ceará	385.644	1021.601	20.683
DALYs	Male	Bahia	457.849	1205.789	26.802
DALYs	Male	Paraná	468.658	1239.547	23.855
DALYs	Male	Rondônia	364.186	944.707	19.504
DALYs	Male	Goiás	419.904	1082.932	22.650
DALYs	Male	Piauí	389.711	1005.112	21.634
DALYs	Male	Distrito Federal	272.457	693.085	14.805

4 - DALYs rates for all ages per 100.000 inhabitants attributable to excessive sodium consumption, female. Brazil, 2019 (95% UI).

				95% UI	
**Measure**	**Sex**	**Location**	**Value**	**Upper**	**Lower**
DALYs	Female	Brazil	204.528	630.394	9.341
DALYs	Female	Minas Gerais	203.191	633.072	8.887
DALYs	Female	Pernambuco	230.124	742.495	9.935
DALYs	Female	Acre	118.819	370.819	5.345
DALYs	Female	Santa Catarina	191.862	585.009	8.243
DALYs	Female	Alagoas	240.992	743.138	10.033
DALYs	Female	Amazonas	103.369	329.261	4.598
DALYs	Female	Mato Grosso	153.840	475.951	6.771
DALYs	Female	Paraíba	222.290	703.631	9.463
DALYs	Female	Maranhão	178.611	547.478	7.696
DALYs	Female	Rio Grande do Norte	178.911	550.384	7.301
DALYs	Female	São Paulo	210.171	667.099	9.253
DALYs	Female	Amapá	106.538	338.860	4.975
DALYs	Female	Roraima	104.093	331.702	4.698
DALYs	Female	Mato Grosso do Sul	196.202	618.474	8.325
DALYs	Female	Pará	130.583	415.914	5.653
DALYs	Female	Sergipe	190.381	595.963	8.084
DALYs	Female	Rio Grande do Sul	248.339	758.134	10.543
DALYs	Female	Tocantins	164.356	514.736	7.053
DALYs	Female	Rio de Janeiro	269.328	845.913	11.644
DALYs	Female	Espírito Santo	222.263	700.645	9.684
DALYs	Female	Ceará	188.954	588.215	7.911
DALYs	Female	Bahia	203.002	643.521	8.396
DALYs	Female	Paraná	219.412	688.747	9.347
DALYs	Female	Rondônia	166.198	526.828	7.208
DALYs	Female	Goiás	189.267	602.948	8.460
DALYs	Female	Piauí	192.748	606.301	8.156
DALYs	Female	Distrito Federal	132.168	418.217	5.868

5 - Age-standardized death rates per 100.000 inhabitants attributable to excessive sodium consumption, male. Brazil, 2019 (95% UI).

				95% UI	
**Measure**	**Sex**	**Location**	**Value**	**Upper**	**Lower**
Deaths	Male	Brazil	19.342	49.242	1.431
Deaths	Male	Pará	18.246	48.055	0.960
Deaths	Male	Mato Grosso do Sul	18.466	48.011	1.061
Deaths	Male	Espírito Santo	21.107	54.985	1.199
Deaths	Male	Maranhão	28.936	76.826	1.620
Deaths	Male	Minas Gerais	15.282	39.988	0.836
Deaths	Male	Rio de Janeiro	21.066	54.469	1.087
Deaths	Male	Amapá	17.742	47.549	0.923
Deaths	Male	Piauí	18.443	47.884	1.034
Deaths	Male	Mato Grosso	15.460	40.578	0.920
Deaths	Male	Ceará	18.883	51.253	0.968
Deaths	Male	Paraíba	19.070	49.344	1.092
Deaths	Male	Pernambuco	23.890	61.788	1.524
Deaths	Male	Tocantins	22.841	61.285	1.239
Deaths	Male	Rondônia	18.117	47.331	0.962
Deaths	Male	Alagoas	23.499	61.254	1.244
Deaths	Male	Roraima	19.996	51.940	1.110
Deaths	Male	Sergipe	18.479	49.496	0.956
Deaths	Male	Acre	20.115	54.056	1.203
Deaths	Male	Goiás	17.998	46.970	0.995
Deaths	Male	Rio Grande do Sul	17.615	44.504	0.996
Deaths	Male	Rio Grande do Norte	17.387	47.055	0.994
Deaths	Male	Bahia	21.659	56.623	1.174
Deaths	Male	Paraná	19.316	50.636	0.974
Deaths	Male	Amazonas	16.820	45.724	0.959
Deaths	Male	São Paulo	20.080	48.826	1.232
Deaths	Male	Santa Catarina	17.148	45.144	0.974
Deaths	Male	Distrito Federal	17.931	46.944	1.025

6 - Age-standardized death rates per 100.000 inhabitants attributable to excessive sodium consumption, female. Brazil, 2019 (95% UI).

Measure	Sex	Location	Value	95% UI Upper	95% UI Lower
Deaths	Female	Brazil	8.736	26.796	0.389
Deaths	Female	Pará	8.100	25.392	0.338
Deaths	Female	Mato Grosso do Sul	9.141	28.283	0.385
Deaths	Female	Espírito Santo	9.711	30.162	0.405
Deaths	Female	Maranhão	10.324	31.605	0.442
Deaths	Female	Minas Gerais	7.620	23.273	0.322
Deaths	Female	Rio de Janeiro	9.431	29.293	0.403
Deaths	Female	Amapá	7.899	24.858	0.341
Deaths	Female	Piauí	9.014	28.742	0.374
Deaths	Female	Mato Grosso	8.555	26.026	0.351
Deaths	Female	Ceará	9.099	27.818	0.372
Deaths	Female	Paraíba	9.394	29.042	0.393
Deaths	Female	Pernambuco	10.585	33.110	0.447
Deaths	Female	Tocantins	9.263	28.618	0.385
Deaths	Female	Rondônia	10.065	30.714	0.411
Deaths	Female	Alagoas	12.463	37.037	0.496
Deaths	Female	Roraima	9.972	30.247	0.417
Deaths	Female	Sergipe	9.371	28.644	0.380
Deaths	Female	Acre	8.734	26.384	0.364
Deaths	Female	Goiás	8.969	27.385	0.387
Deaths	Female	Rio Grande do Sul	8.706	26.398	0.365
Deaths	Female	Rio Grande do Norte	7.860	24.195	0.308
Deaths	Female	Bahia	8.667	27.546	0.345
Deaths	Female	Paraná	9.519	29.035	0.392
Deaths	Female	Amazonas	7.390	23.377	0.315
Deaths	Female	São Paulo	8.122	25.207	0.353
Deaths	Female	Santa Catarina	8.867	26.479	0.370
Deaths	Female	Distrito Federal	9.328	28.165	0.391

7 - Age-standardized DALYs rates per 100.000 inhabitants attributable to excessive sodium consumption, male. Brazil, 2019 (95% UI).

				95% UI	
**Measure**	**Sex**	**Location**	**Value**	**Upper**	**Lower**
DALYs	Male	Brazil	434.134	1092.624	30.861
DALYs	Male	Minas Gerais	357.245	929.549	17.998
DALYs	Male	Pernambuco	549.287	1410.613	32.874
DALYs	Male	Acre	410.312	1076.269	22.940
DALYs	Male	Santa Catarina	369.702	963.968	20.138
DALYs	Male	Alagoas	547.097	1428.462	27.590
DALYs	Male	Amazonas	367.753	987.305	20.638
DALYs	Male	Mato Grosso	354.333	904.718	20.349
DALYs	Male	Paraíba	440.039	1109.713	25.780
DALYs	Male	Maranhão	598.416	1589.679	32.464
DALYs	Male	Rio Grande do Norte	394.204	1060.709	22.301
DALYs	Male	São Paulo	447.235	1079.722	26.687
DALYs	Male	Amapá	390.539	1031.894	19.917
DALYs	Male	Roraima	405.614	1022.224	22.781
DALYs	Male	Mato Grosso do Sul	416.141	1065.367	23.907
DALYs	Male	Pará	407.244	1066.126	21.558
DALYs	Male	Sergipe	413.949	1092.735	22.321
DALYs	Male	Rio Grande do Sul	384.564	967.531	19.410
DALYs	Male	Tocantins	464.008	1217.349	24.161
DALYs	Male	Rio de Janeiro	491.833	1226.672	25.654
DALYs	Male	Espírito Santo	463.990	1180.964	25.748
DALYs	Male	Ceará	415.412	1109.829	21.653
DALYs	Male	Bahia	486.401	1274.438	28.530
DALYs	Male	Paraná	419.437	1098.956	21.506
DALYs	Male	Rondônia	402.207	1050.498	21.407
DALYs	Male	Goiás	414.270	1070.445	23.132
DALYs	Male	Piauí	405.903	1045.633	22.509
DALYs	Male	Distrito Federal	343.669	884.004	19.906

8 - Age-standardized DALYs rates per 100.000 inhabitants attributable to excessive sodium consumption, female. Brazil, 2019 (95% UI).

				95% UI	
**Measure**	**Sex**	**Location**	**Value**	**Upper**	**Lower**
DALYs	Female	Brazil	174.379	536.769	7.983
DALYs	Female	Minas Gerais	156.349	488.977	6.874
DALYs	Female	Pernambuco	212.258	682.002	9.155
DALYs	Female	Acre	170.222	524.142	7.476
DALYs	Female	Santa Catarina	159.706	486.468	6.872
DALYs	Female	Alagoas	257.974	790.590	10.638
DALYs	Female	Amazonas	145.514	464.253	6.347
DALYs	Female	Mato Grosso	167.141	509.098	7.259
DALYs	Female	Paraíba	188.727	599.078	8.042
DALYs	Female	Maranhão	208.947	638.607	8.923
DALYs	Female	Rio Grande do Norte	158.120	486.282	6.406
DALYs	Female	São Paulo	161.980	515.922	7.161
DALYs	Female	Amapá	162.437	515.204	7.329
DALYs	Female	Roraima	174.935	545.810	7.708
DALYs	Female	Mato Grosso do Sul	181.831	567.714	7.741
DALYs	Female	Pará	165.568	524.696	7.043
DALYs	Female	Sergipe	188.781	590.054	7.994
DALYs	Female	Rio Grande do Sul	165.419	509.987	7.085
DALYs	Female	Tocantins	184.452	580.641	7.841
DALYs	Female	Rio de Janeiro	193.509	605.834	8.438
DALYs	Female	Espírito Santo	189.526	596.656	8.247
DALYs	Female	Ceará	176.784	550.106	7.375
DALYs	Female	Bahia	183.650	587.236	7.550
DALYs	Female	Paraná	178.483	557.590	7.644
DALYs	Female	Rondônia	192.896	605.611	8.194
DALYs	Female	Goiás	178.302	559.093	7.953
DALYs	Female	Piauí	176.802	559.588	7.476
DALYs	Female	Distrito Federal	152.39	474.948	6.691
